# Synthesizability *via* reward engineering: expanding generative molecular design into synthetic space

**DOI:** 10.1039/d5sc09263a

**Published:** 2026-04-07

**Authors:** Dominik Dekleva, Alexey Voronov, Jon Paul Janet, Albin Ekborg, Jure Borišek, Martina H. Rambaher, Hannes H. Loeffler

**Affiliations:** a National Institute of Chemistry Hajdrihova 19 Ljubljana 1000 Slovenia dominik.dekleva@ki.si; b Faculty of Pharmacy, University of Ljubljana Ljubljana Slovenia; c Molecular AI, Discovery Sciences, BioPharmaceuticals R&D, AstraZeneca Gothenburg Sweden; d Department of Physics, Chalmers University of Technology Gothenburg Sweden

## Abstract

Generating novel, drug-like molecules with realistic synthetic pathways is an essential goal in computer-aided drug discovery, yet generative models often lack synthesis awareness, resulting in compounds that are difficult or impossible to produce. To overcome this limitation, models must optimize not only molecular properties but also synthetic feasibility, which is not fully meaningful unless it incorporates specific factors like preferred reactions and available starting materials. Moreover, generating singleton compounds without respecting possibilities for parallel synthesis greatly increases the cost and complexity of synthesizing multiple proposed molecules. In practice, medicinal chemistry workflows group compounds into families sharing coherent synthetic strategies and common intermediates, enabling efficient parallel and automated synthesis. Here we introduce SynthSense, a reinforcement learning framework that guides molecular design using retrosynthetic feedback. SynthSense offers extrinsic reward functions that assess molecule-level feasibility, such as adherence to available building blocks and preferred reactions, or synthesizability *via* predefined synthetic routes. It also implements intrinsic, batch-level functions that enforce route coherence across generated compounds. *In silico* multi-parameter validation demonstrated clear advantages over naïve, synthesis-unaware baselines: SynthSense generated 6.2-fold more synthetically feasible hits than control trained without SynthSense, achieved a 727-fold enrichment in hits synthesizable with a predefined synthetic route, and populated 4.1-fold more virtual parallel synthesis plates. These results demonstrate that by reframing synthesizability from a mere constraint into an active design objective, generative AI can better support the realities of modern medicinal chemistry by enabling personalized synthetic design, accelerating SAR exploration and aligning more naturally with automated parallel synthesis workflows.

## Abstract 2

SynthSense couples generative molecular AI with retrosynthetic reward functions to produce novel, drug-like molecules that are not only feasible to synthesize but aligned with the parallel synthesis workflows of modern medicinal chemistry.

## Graphical abstract

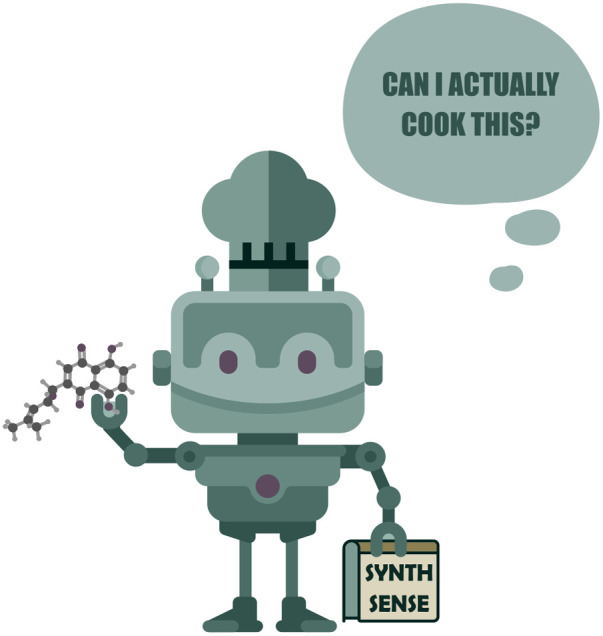

## 1 Introduction

Discovering novel, drug-like molecules is a cornerstone of pharmaceutical innovation, yet the scale of chemical space, estimated to contain over 10^60^ drug-like compounds, makes exhaustive experimental exploration impossible.^1^ Recently, deep generative models have emerged as powerful tools for exploring this vast landscape, enabling *de novo* design of molecules with tailored properties.^2^ Approaches such as variational autoencoders,^3^ diffusion models,^4^ and reinforcement learning (RL)-driven frameworks^5,6^ have proven effective in generating chemically valid, diverse, and property-optimized compounds.

Many proposed structures, while optimal *in silico*, are difficult or impossible to synthesize in practice.^7^ To close this gap between computational design and laboratory realization, several methods to incorporate synthetic accessibility (SA) into the molecular generation process have been developed. Early efforts produced heuristic metrics, most notably the Synthetic Accessibility Score^8^ and machine learning (ML) classifiers, such as the Synthetic Complexity Score,^9^ which estimate the ease of synthesis based on molecular complexity and fragment occurrence. While useful, these metrics are limited by their reliance on historical data and inability to account for the dynamic, context-dependent nature of chemical synthesis.

The advent of computer-aided synthesis planning (CASP) tools, such as AiZynthFinder,^10^ ASKCOS,^11^ and LillyMol,^12^ has enabled more rigorous, retrosynthesis-based assessment of synthetic routes. These platforms leverage reaction databases and ML to propose stepwise synthetic pathways from in-house or commercially available starting materials to target molecules.

Building on these advances, the Retrosynthetic Accessibility Score (RAscore)^13^ was developed as an ML classifier that rapidly predicts whether a retrosynthetic route can be identified for a given molecule by AiZynthFinder. While RAscore is well-suited for pre-screening large virtual libraries for synthetic feasibility and can serve as a reward function within generative frameworks such as REINVENT,^6^ it represents a static approximation trained on AiZynthFinder output with a fixed starting materials stock configuration.

There is growing interest in synthesis-aware molecular generation, where feedback from synthesis planning is incorporated directly into the generative process.^14–21^ Such approaches can enable the generative policy to adapt dynamically to synthetic constraints with route-level precision, going beyond heuristic and structure-based SA scores, which offer a coarser and often less reliable proxy for synthetic feasibility.

Most current synthesis-aware approaches (*e.g.*, SynFlowNet,^15^ PrexSyn,^16^ ReaSyn,^17^ SynLLaMA^18^) integrate synthetic constraints during model training, requiring specialized architectures or training procedures tailored to incorporate synthesis planning feedback. While effective, these methods are limited to specific model architectures and cannot easily adapt to dynamic changes in reagent availability, reaction feasibility, or preferred synthetic strategies that occur regularly in real-world medicinal chemistry settings. In contrast, our strategy incorporates synthesis awareness at the post-training stage through RL, treating synthesizability as a REINVENT objective; pre-trained generative models can be used without architectural modifications or retraining requirements.

Previously developed approaches all consider the specific synthesizability of single compounds generated by the model, but this singleton approach, where each molecule is treated in isolation and does not share an obvious synthetic route with any other generated molecule except by chance, poorly reflects how synthesis is executed in practice. Different chemists and project teams also naturally develop preferences and experience with specific reaction types and route archetypes, with condition optimization and existing intermediates meaning certain synthetic routes are more time and resource efficient than others.^22^

Generating singleton compounds without respecting possibilities of parallel synthesis also greatly increases the costs and complexity of synthesizing multiple proposed molecules, whereas in practice molecular targets for synthesis are regularly grouped into sets based on related chemistry.^23,24^ This consideration is important for bespoke synthesis in traditional medicinal chemistry settings, but it becomes especially critical in the context of synthesis automation and robotic platforms,^25,26^ which can perform hundreds of identical synthetic steps in a plate-wise fashion. In this context, synthetic route coherence, the extent to which generated molecules can share common routes, reactions, intermediates, or conditions, is a key determinant of experimental feasibility and cost-effectiveness, and thus essential for practical synthesis strategy.

Therefore, molecular generators should produce not singleton compounds, but families of molecules with coherent synthetic strategies that can be aligned with existing, successful and parallelizable synthetic routes. This shift from optimizing individual molecules to designing synthetic families represents a fundamental reframing of the synthesis-awareness problem to better reflect the realities of modern medicinal chemistry workflows.

**Fig. 1:**
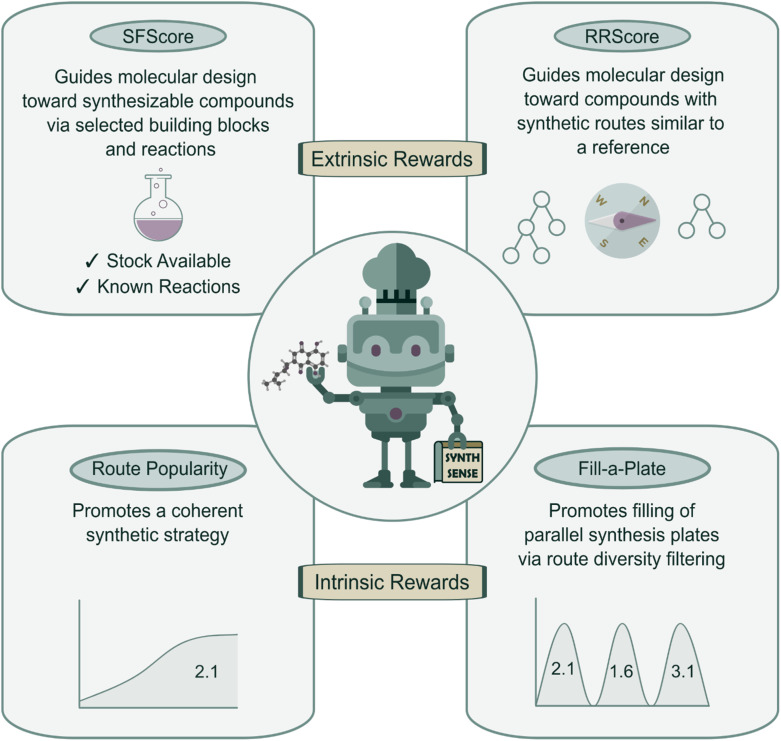
Overview of SynthSense. Four complementary reward functions guide molecular generation toward synthesizable compounds, with Synthetic Feasibility Score (SFScore) and Reference Route Score (RRScore) providing extrinsic rewards for synthetic feasibility and route targeting, while Route Popularity and Fill-a-Plate offer intrinsic rewards for strategic route coherence and filling of parallel synthesis plates.

In this work, we introduce SynthSense (Fig. 1), a novel REINVENT scoring component, which combines intrinsic (batch-level) and extrinsic (single-molecule) reward functions to address the challenge of designing molecules with favorable properties and synthetically feasible and coherent routes. Extrinsic reward functions operate at the single-molecule level and bias generation toward synthetically accessible compounds: Synthetic Feasibility Score (SFScore) evaluates each molecule against user-specified criteria such as available starting materials and preferred reactions, while the Reference Route Score (RRScore) steers molecular design toward compounds synthesizable *via* a predefined synthetic reference route. Intrinsic reward functions operate at the batch level and shape the collective behavior of the generator: Route Popularity promotes coherent synthetic strategies within batches of generated molecules, whereas Fill-a-Plate autonomously balances exploitation and exploration of synthetic space to encourage filling of virtual parallel synthesis plates.

In parallel to our work, Guo *et al.*^20^ introduced an extrinsic reward function conceptually related to our SFScore that enforces or avoids specific reactions *via* binary reward multipliers applied to predicted routes. In contrast, SFScore is a continuous, floating-point route feasibility score derived from retrosynthesis that jointly captures stock availability, reaction-class membership, and route complexity.

With SynthSense, the generative model can prioritize synthetic routes in real time, explore novel pathways or exploit established synthetic strategies. We validate SynthSense *in silico*, showing that its rewards yield 6.2-fold more synthetically feasible hits, a 727-fold enrichment in molecules synthesizable with a predefined synthetic route, and a 4.1-fold increase in virtual parallel synthesis plate coverage compared to control trained without SynthSense. These results highlight how retrosynthesis-driven rewards can improve the practical application of generative models in drug discovery.

## 2 Methods

SynthSense guides molecular design across synthetic space by integrating retrosynthetic feedback into RL, leveraging AiZynthFinder for retrosynthetic analysis and NameRxn^27^ for reaction classification. In its current implementation, SynthSense is integrated as a plug-and-play reward family for REINVENT built on top of AiZynthFinder and thus inherits the model and catalog biases of this stack, although the same concepts could in principle be ported to other RL molecular generators and tree-based CASP tools.

For each generated molecule, AiZynthFinder enumerates all possible synthetic routes from available starting materials. Routes that successfully trace back to stock reagents are considered solved, whereas partially resolved retrosynthetic trees are retained as unsolved routes. Different rewards make use of solved and unsolved routes differently: RRScore and Route Popularity compute rewards exclusively from solved routes, whereas SFScore and Fill-a-Plate incorporate both solved and unsolved routes.

For SFScore, Route Popularity, and Fill-a-Plate, routes are converted into route signatures by encoding each reaction step using the NameRxn reaction taxonomy, which classifies reactions hierarchically into superclass (*X*), class (*XY*), and named reaction (*XYZ*). SFScore evaluates signatures at the named-reaction level (*XYZ*) for fine-grained control over specific reactions, whereas Route Popularity and Fill-a-Plate operate at the broader reaction-class level (*XY*), allowing reaction diversity within parallel synthesis plates.

In RRScore, routes are instead represented as retrosynthetic trees annotated with NameRxn reaction labels at the named-reaction level (*XYZ*), and the reward is computed *via* Tree Edit Distance (TED) to quantify similarity to a reference route tree.

To demonstrate SynthSense capabilities in a realistic drug discovery context, we ran multi-parameter optimization (MPO) RL experiments with REINVENT, combining synthesis awareness with established drug design objectives, including drug-likeness,^28^ and ligand-based shape similarity.^29–33^ Drug-likeness, a qualitative measure of how “drug-like” a molecule is in terms of physicochemical and pharmacokinetic properties, was estimated using the Quantitative Estimate of Drug-likeness (QED)^34^ metric, which integrates multiple molecular descriptors into a continuous score between 0 and 1.

Rapid Overlay of Chemical Structures (ROCS)^30–33^ was used as a ligand-based reward function to evaluate 3D similarity between generated molecules and a reference ligand. The method assesses both molecular shape and pharmacophoric (“color”) overlap, yielding a Tanimoto Combo score between 0 and 1 as implemented in REINVENT.^6^

COX-2 inhibitor SC-558 (PDB ID: 1CX2, structure shown in Fig. S1)^35^ was used as the reference ligand and a ROCS query was derived from its crystallographic binding pose. The self-overlay of SC-558 yielded a Tanimoto Combo score of 0.6, which we used as the similarity threshold for identifying hits. Full details of the ROCS setup, conformer generation, and query feature definitions are provided in the (SI).

### 2.1 Biasing toward synthetically feasible molecules

The SFScore is an extrinsic reward that quantifies SA by evaluating how readily a molecule can be synthesized using available starting materials, preferred reactions and step limits. This guided approach addresses the limitations of traditional SA scores by incorporating dynamic, user-defined constraints that better reflect real-world medicinal chemistry workflows, where factors like reagent availability and reaction preferences vary by project or lab. A composite score reflecting all three factors is calculated for each synthetic tree and a molecule is scored as its highest scored tree. The SFScore score for a single tree is computed as the product of three components: Stock Availability Score, Reaction Score and Step Score.

Stock Availability Score evaluates precursor accessibility based on commercial or in-house availability. By default, precursors from internal stocks receive a score of 1.0, commercially available compounds score 0.8, and unavailable precursors score 0.1. The total stock score represents the product of all individual precursor stock scores within a tree.

Reaction score assesses the reaction preference by scoring individual reactions. By default, specified preferred reactions receive a score of 1.0, while others are scored 0.1. The total reaction score is the product of individual reaction scores in each tree.

Step score penalizes synthetic complexity using an exponential decay function *k*^*n*^, where *k* is the reaction step coefficient (default 0.9) and *n* represents the number of synthetic steps in the tree. For stock-available compounds (*n* = 0), the score equals 1.0, while a three-step synthesis (*n* = 3) yields 0.9^3^ = 0.729 and so on.

SFScore ensures that the score degrades appropriately when any component becomes limiting, reflecting the practical constraints of medicinal chemistry synthesis.

Let *T*_i_ be the set of all synthetic trees for molecule *i*. For any tree *t* ∈ *T*_i_ we define three factors:

1. Stock Availability Score
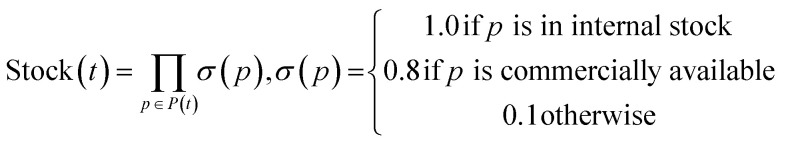
where *P*(*t*) denotes the set of precursor molecules required in synthetic tree *t*.

2. Reaction score
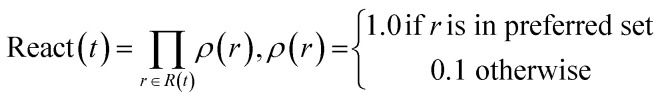
where *R*(*t*) denotes the set of reactions required in synthetic tree *t*.

3. Step scoreStep(*t*) = *k*^*n*(*t*)^,0 < *k* < 1,*n*(*t*) = number of reaction steps in tree *t*

The SFScore score for tree *t* is the product of these factors:*S*(*t*) = Stock(*t*) *x* React(*t*) *x* Step(*t*).

The final score for molecule *i* is taken as its highest-scoring tree:
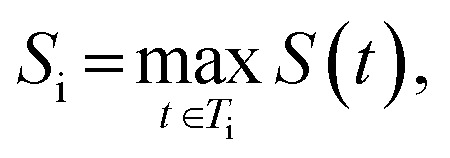


so higher values of *S*_i_ indicate molecules whose best synthetic route is easy to realize.

### 2.2 Biasing toward target synthetic routes

**Fig. 2:**
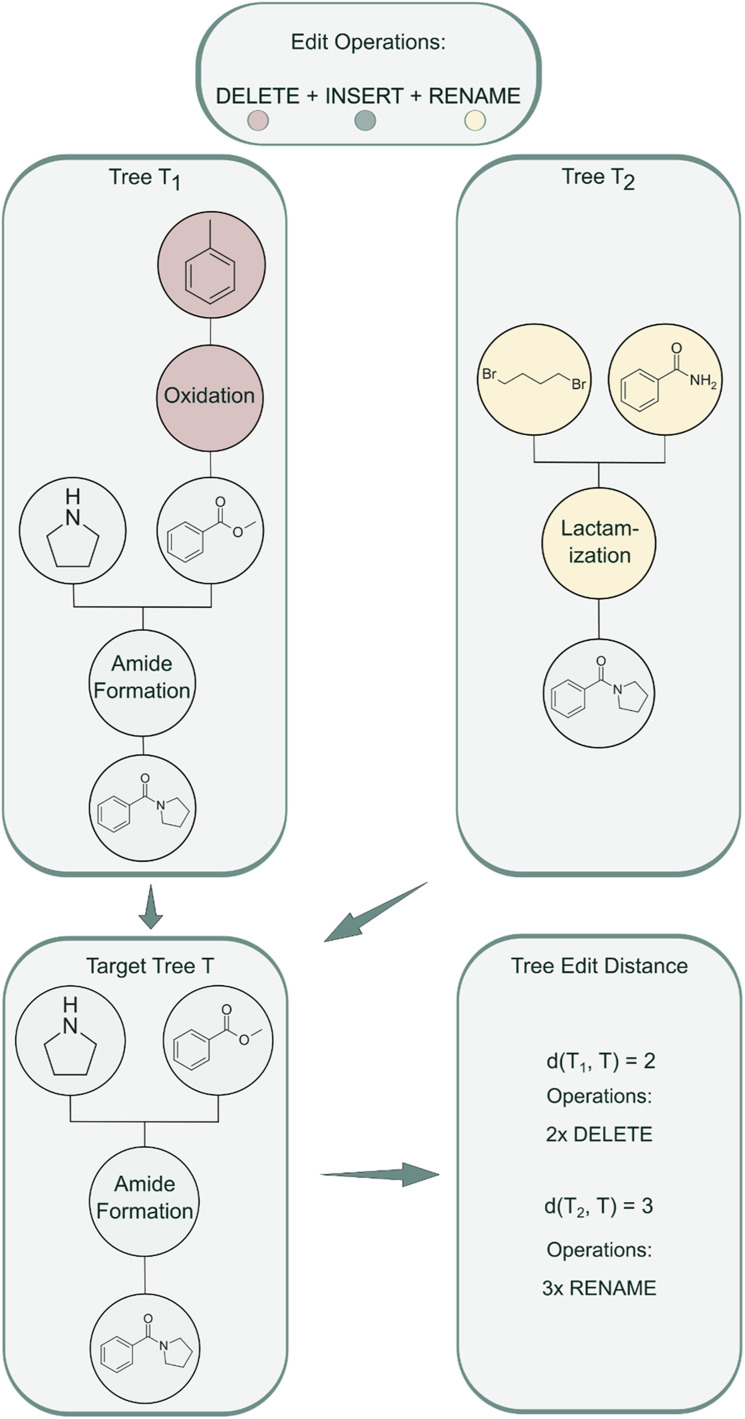
TED algorithm applied to molecular reaction pathways. Two input synthesis trees (*T*_1_ and *T*_2_) are transformed into a target tree (*T*) using edit operations.

RRScore is an extrinsic reward that guides molecular generation toward compounds synthesizable *via* a predefined reference synthetic route by quantifying the similarity between a generated molecule's synthetic tree and the reference. Similarity is measured using TED, which calculates the minimum number of edit operations (delete, insert, rename) required to transform one tree into another (Fig. 2).

We implemented TED using the apted python package,^36,37^ with node deletion and insertion costs of 4.0, while rename operations employ hierarchically scaled costs: NameRxn superclass differences (1.*x vs.* 2.*x*) cost 3.0, class-level differences (2.1 *vs.* 2.2) cost 2.0, and named reaction differences cost 1.0. This TED configuration provides fine-grained distance metrics useful for gradually steering design towards a target synthetic route.

Decreasing TED values indicate convergence toward the reference synthetic pathway, reflecting the function's ability to guide generation toward the target route.

Let *T*_i_ be the set of solved synthetic trees for molecule *i*. If no valid synthetic tree is found (*i.e.*, *T*_i_ is empty), the RRScore for that molecule is assigned a value of 0.

1 Tree distanced(*t*) = TED(*t*, *t*_ref_)(*t* ∈ *T*_i_)

2 Similarity for each tree
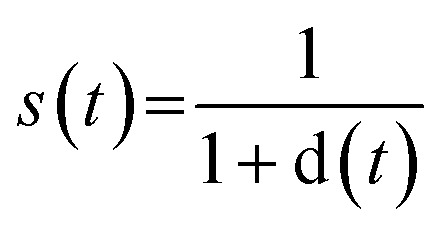


3 RRScore for molecule *i* is the maximum similarity across all solved trees:
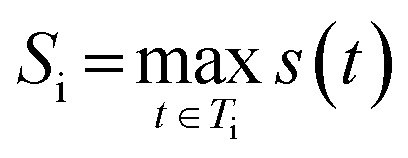
where *S*_i_ = 1 for an identical route (TED = 0 under our cost scheme) and declines towards 0 as the pathways diverge.

While one could, in principle, perform reaction-based enumeration around a fixed reference route to obtain additional candidates, such an approach is limited to varying reagents and then post-filtering for properties of interest. In contrast, our generative RL formulation with RRScore directly optimizes an MPO signal (route similarity, shape similarity, QED) within a single policy, allowing synthesizability and property-based objectives to be balanced during generation rather than imposed as a separate, downstream filtering step.

### 2.3 Biasing toward popular synthetic routes

Route Popularity is an intrinsic reward function that leverages NameRxn reaction classes to measure the extent to which specific synthetic strategies are shared among molecules within a batch. This rewards the emergence of common synthetic approaches, which can be valuable for identifying robust, generalizable routes and for promoting batch-level synthetic coherence.

**Fig. 3:**
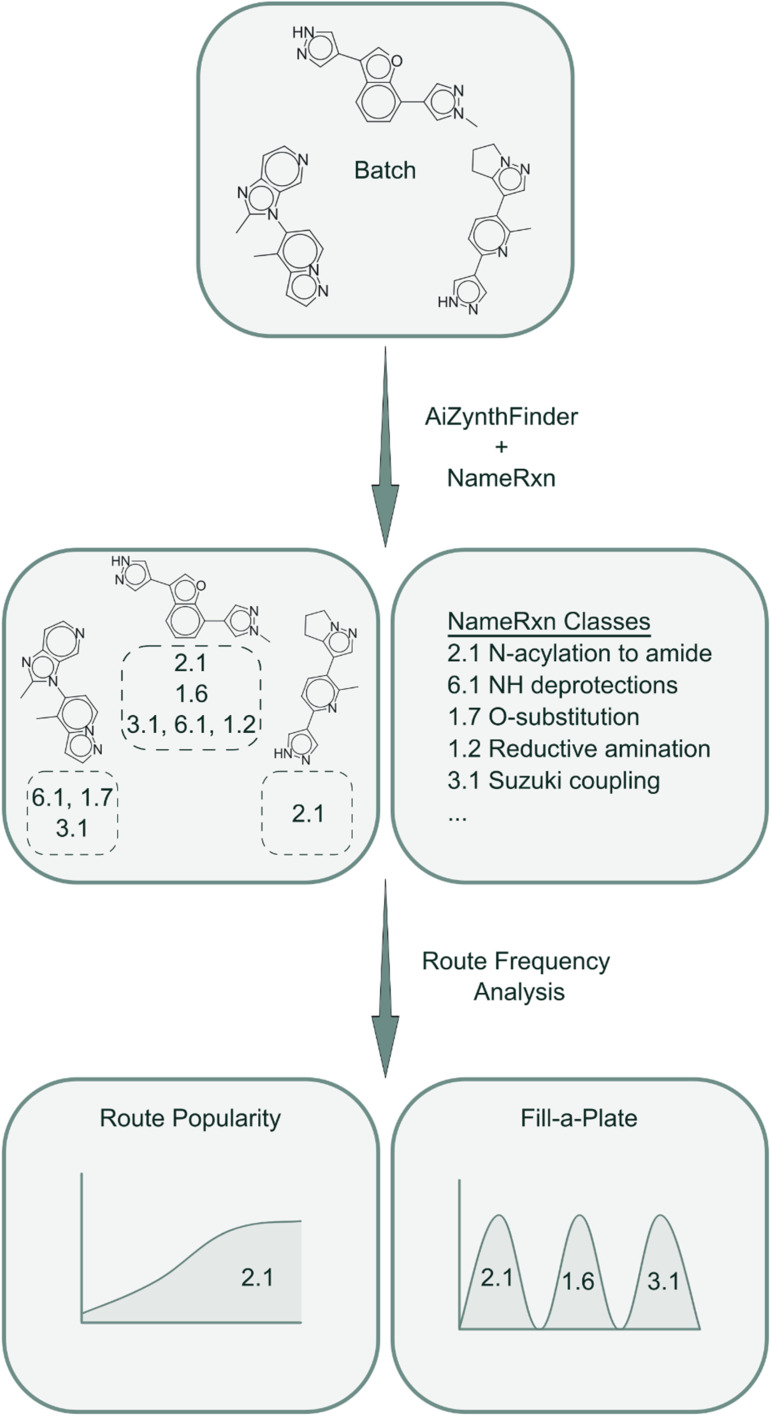
Overall workflow of Route Popularity and Fill-a-Plate reward functions. A batch of generated molecules undergoes retrosynthetic analysis with AiZynthFinder, followed by NameRxn reaction classification to extract route signatures. For Route Popularity (left), intra-batch route frequencies are calculated to optimize for molecules sharing common routes; for Fill-a-Plate (right), a route diversity filter tracks frequencies across batches, filling virtual parallel synthesis plates and penalizing saturated routes to pivot toward novel strategies, thereby promoting balanced exploration and exploitation of synthetic space.

To capture shared synthetic strategies, we first encode each solved synthetic route generated by AiZynthFinder as a route signature, defined as the set of NameRxn reaction classes present in the route (Fig. 3). This signature serves as a unique identifier for the synthetic strategy employed.

For each unique route signature observed in the batch, we count the number of distinct molecules that possess a route with this signature. This count is then normalized by the total number of molecules in the batch to yield the molecule popularity of the route.

For each molecule, the Route Popularity score is determined as the maximum molecule popularity among all of its solved trees, thereby rewarding molecules that utilize widely applicable synthetic strategies within a batch.

Let *S*_t_ denote the route signature of route tree *t*, and let *M*_s_ be the set of molecules in the batch that have a solved tree with signature *S*. The molecule popularity of signature *S* is given by:
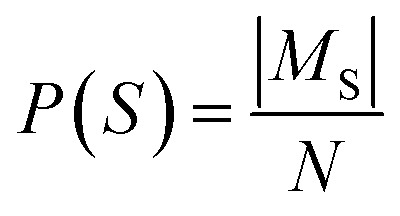
where *N* is the number of all molecules in the batch. The popularity score for tree *t* is then:
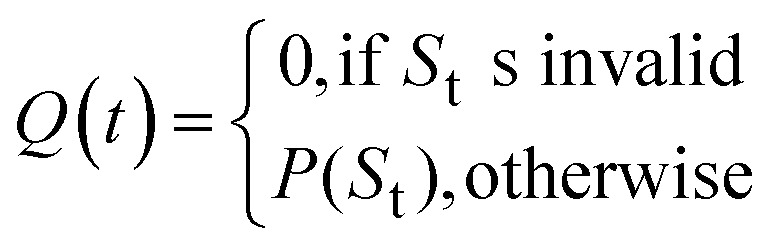


The final Route Popularity score for molecule *i* is:
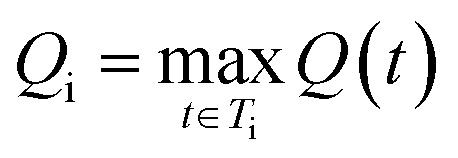
where *T*_i_ is the set of solved trees for molecule *i*. Invalid signatures (*e.g.*, containing unrecognized reactions) receive a score of 0.

*Q*_i_ value of 1 indicates that molecule *i* shares a popular synthetic route with all other molecules in the batch, while lower values toward 0 reflect the use of less common or unique synthetic strategies.

### 2.4 Biasing toward filling parallel synthesis plates

The Fill-a-Plate is an intrinsic reward which implements a route diversification strategy that builds upon Route Popularity by incorporating a dynamic route diversity filter.^38^ This enhanced approach balances the exploitation and exploration of synthetic space through cumulative tracking and penalization mechanisms, maintaining a memory of route usage across batches. The method was originally conceived to address the practical need for populating parallel synthesis plates in high-throughput experimental workflows, hence the name “Fill-a-Plate”.

For each synthetic tree generated by AiZynthFinder, a route signature is extracted using NameRxn to classify reaction steps (Fig. 3). The Fill-a-Plate algorithm tracks the cumulative number of unique molecules associated with each route signature across batches. For every generated molecule, the algorithm scans all of its associated route signatures and identifies the one closest to the predefined plate threshold (*e.g.*, 1000 molecules per route) and assigns the molecule a reward proportional to that signature's filling ratio (*e.g.*, 0.7 for a route that is 70% full). Once a route reaches capacity, its signature is no longer considered for scoring in subsequent epochs, and molecules that contributed to filling it can optionally also be excluded from future plates to prevent recycling. This design encourages the model to exploit productive routes up to a point but is then pushed to explore alternative synthetic solutions once a route becomes saturated.

In this work, we implemented the optional molecule exclusion to demonstrate that the algorithm can populate multiple plates without reusing molecules, pivoting in both synthetic and chemical space.

Let *S*_t_ denote the route signature of tree *t* and let *c*_St_ be the cumulative count of unique molecules associated with signature *S*_t_ across all batches. Let *C* be the plate capacity threshold. The score for tree *t* is given by:
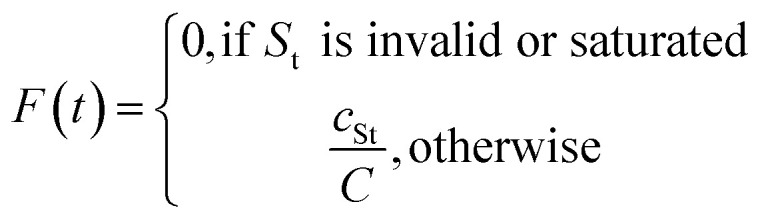


The final score for molecule *i* is:
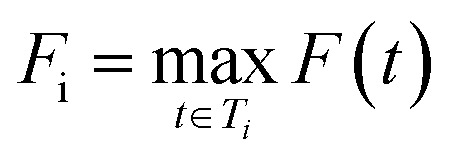
where *T*_i_ is the set of all trees for molecule *i*. Higher values of *F*_i_ indicate that molecule *i* is associated with a route that is being actively exploited but has not yet reached its capacity, while a score of zero reflects either saturation or the use of an invalid route that contains unrecognized reactions.

### 2.5 Experimental setup

All RL experiments were conducted in triplicates using REINVENT with the classical RNN-based *de novo* REINVENT prior, which is trained to mimic the ChEMBL database.^39^ Training was run with a batch size of 128 for 1000 epochs.

For SFScore, reaction space was constrained to 3 NameRxn reaction classes: Suzuki coupling reactions (3.1), amide formation reactions (2.1), and *N*-arylations (1.3), with a maximum of 3 synthetic steps per route. Feasible hits were defined as molecules synthesizable from those reaction classes using 313279 Enamine Building Blocks (EU stock) as starting materials and exceeding ROCS > 0.6 (relative to the native COX-2 inhibitor) and QED > 0.7, establishing a pharmaceutically relevant benchmark for synthesis-aware molecular design.

For RRScore, we used a fixed two-step reference route (2.1.10 Carboxylic ester + amine reaction, 3.1.2 Chloro Suzuki coupling). Molecules were classified as reference route hits if they could be synthesized *via* this route from 313279 Enamine Building Blocks (EU stock), while also exceeding QED > 0.7 and ROCS > 0.6. This route was chosen to provide a simple, yet representative example of a drug-like synthetic sequence.

For Route Popularity, we analyzed the evolution of route utilization across training runs. The fraction of molecules within batches following each route signature was tracked across epochs, providing a measure of how the model's synthetic preferences evolved over time. We focused this analysis on the five most frequently used routes. In addition, we tracked the accumulation of all generated molecules, hits and their scaffolds within these routes to assess hit discovery.

In case of Fill-a-Plate, plate capacity was set to 1000, meaning that each plate corresponds to a maximum of 1000 molecules sharing the same route signature.

Route Popularity and Fill-a-Plate hits were defined as molecules synthesizable in maximum 3 steps from 313279 Enamine Building Blocks (EU stock)while also exceeding QED > 0.7 and ROCS > 0.6.

In all experimental runs, RL optimization was performed against a geometric mean reward comprising SynthSense, QED, and ROCS, each with equal weight. In the control runs, the weight of SynthSense was set to 0. Thus, SynthSense rewards were still evaluated to log synthetic metrics for comparison but did not contribute to the MPO score.

## 3 Results

All rewards were set up using both solved-only retrosynthesis trees, and the combined solved and unsolved configuration (as defined in Methods). For each reward, we report results using the configuration that performed the best: RRScore and Route Popularity use solved routes only, whereas SFScore and Fill-a-Plate operate on both solved and unsolved routes. MPO and chemical diversity plots for all rewards are provided in the SI.

### 3.1 Biasing toward synthetically feasible molecules

**Fig. 4:**
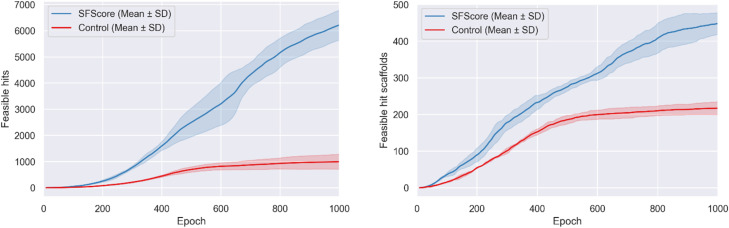
Accumulation of unique feasible hits (left) and hit scaffolds (right) identified by REINVENT with and without the SFScore reward function. Each line represents the mean over three independent training runs (± standard deviation). The inclusion of SFScore accelerates the generation of both synthetically feasible hits and hit scaffolds compared to the control.

The superior performance of SFScore *vs.* control was validated by cumulative feasible hit accumulation curves showing sustained linear growth without saturation (Fig. 4).

SFScore generated 6237 ± 572 feasible hits compared to 1000 ± 290 for the control (∼6.2-fold improvement), maintaining consistently higher feasible hit generation rates throughout the optimization process, particularly after epoch 200 when the model learned to effectively navigate synthetically feasible chemical space.

The synthesis-aware approach also demonstrated substantially greater feasible hit scaffold accumulation (Fig. 4), generating 449 ± 31 unique feasible hit scaffolds compared to 218 ± 18 for the control (∼2.1-fold improvement).

### 3.2 Biasing toward target synthetic routes

**Fig. 5:**
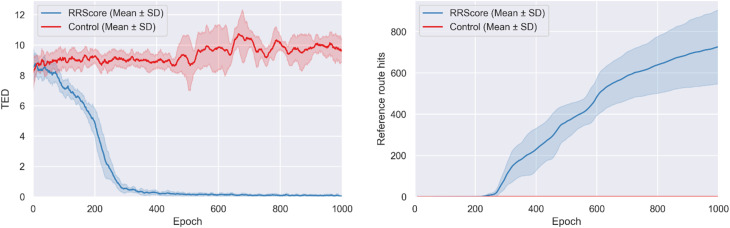
Training progression with the RRScore reward compared to the control. The left panel shows the decrease in average Tree Edit Distance (TED) between generated and reference synthetic routes, indicating convergence toward the target pathway. The right panel shows the accumulation of reference route hits. Each curve represents the mean across three independent runs (± standard deviation). RRScore optimization produced a substantial reduction in TED and generated significantly more reference route hits than the control.

During training, molecules optimized with the RRScore showed a clear convergence toward the reference route (2.1.10 Carboxylic ester + amine reaction, 3.1.2 Chloro Suzuki coupling), with the average TED decreasing from 8.5 ± 1.3 at the start to 0.05 ± 0.06 (Fig. 5). In contrast, control remained near their initial TED values and even slightly diverged, increasing from 8.2 ± 1.2 to 9.7 ± 0.8. This demonstrates that RRScore strongly biases design toward the target synthetic pathway while control shows no such convergence.

RRScore optimization produced 727 ± 179 reference route hits compared to just 1 for the control (∼727-fold improvement), demonstrating a dramatic enrichment in molecules aligned with the desired synthetic pathway (Fig. 5). In terms of structural diversity, RRScore also achieved 22.3 ± 2.5 unique reference route hit scaffolds, whereas the control remained flat at 1.0 (∼22.3-fold improvement, scaffold plot in Fig. S2).

### 3.3 Biasing toward popular synthetic routes

**Fig. 6:**
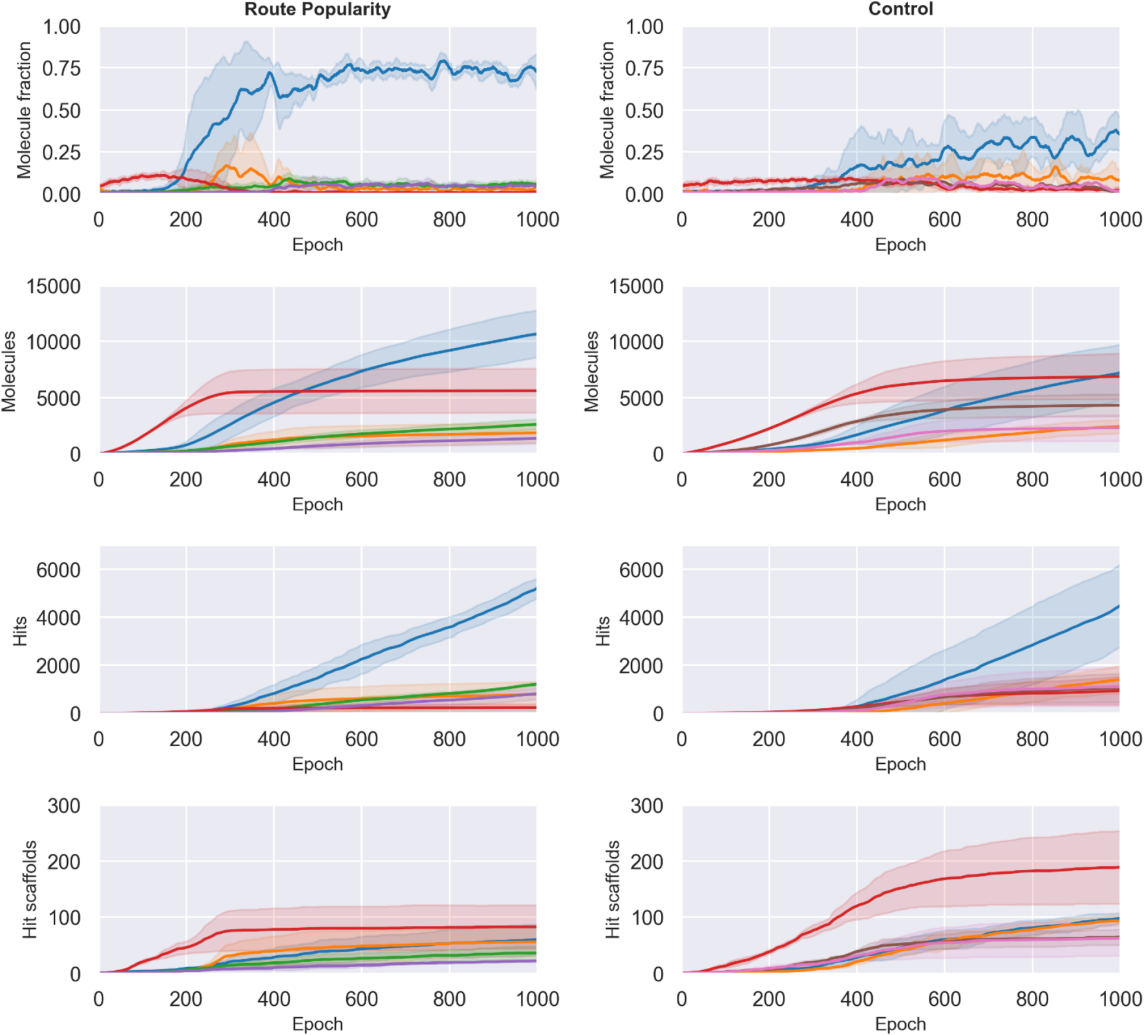
Impact of the Route Popularity reward on generative behavior compared to the control. Top row shows the fraction of each batch that is synthesizable *via* the top five most common route signatures, revealing rapid convergence toward a single dominant synthetic pathway under Route Popularity. Second row reports the cumulative number of unique generated molecules accessible through these same routes, while the third and fourth rows show cumulative hit and hit scaffold accumulation, respectively. Curves indicate mean performance across three independent runs (± standard deviation). Route Popularity strongly enriches batch-level route coherence but yields only modest improvements in unique molecule and hit discovery relative to the control.

The Route Popularity reward rapidly concentrated generation toward a dominant single-step 1.8 S-substitution route, increasing its batch fraction from 0.009 ± 0.003 to 0.706 ± 0.159 by epoch 1000, whereas the control reached 0.339 ± 0.143 (∼2.1-fold higher, Fig. 6, top row). Other major routes such as single-step *N*-acylation to amide (2.1) declined (0.011 ± 0.007 *vs.* 0.022 ± 0.006; ∼2.0-fold lower), demonstrating that the reward drives strong convergence onto a narrow synthetic subspace.

However, this large per-batch shift did not translate proportionally into discovery of new molecules. As seen in second row of Fig. 6, the cumulative number of unique generated molecules accessible *via* route 1.8 was only slightly higher for Route Popularity (10 696 ± 2097) compared with control (7232 ± 2507). This mismatch indicates substantial regeneration of duplicates, reflecting over-exploitation of a narrow synthetic (and chemical) pocket.

Hit discovery followed a similar same trend: Route Popularity yielded 5273 ± 366 hits *vs.* 4549 ± 1735 in control (∼1.2-fold increase, Fig. 6, third row), a weaker improvement than expected given the dramatic enrichment in batch composition. This effect also extended to structural novelty: despite more hits overall, Route Popularity generated fewer unique hit scaffolds (60 ± 27 *vs.* 99 ± 9; ∼1.6-fold lower; Fig. 6, bottom row), reinforcing that hit-level diversity suffers when the search space narrows. These results suggest that while Route Popularity successfully biases generation toward synthetically favorable chemistry and yields actionable route coherence, it does not improve the model's ability to find better hits. Control appears to benefit from broader exploration, accumulating generalizable SAR knowledge that Route Popularity restricts.

### 3.4 Biasing toward filling parallel synthesis plates

Building on Route Popularity, the Fill-a-Plate reward introduces a route diversification mechanism that penalizes saturated synthetic routes while rewarding molecules that contribute to under-filled plates. By design, this counteracts the collapse of Route Popularity onto a single synthetic pocket, creating dynamic pressure to pivot into new regions of synthetic space as plates reach capacity.

**Fig. 7:**
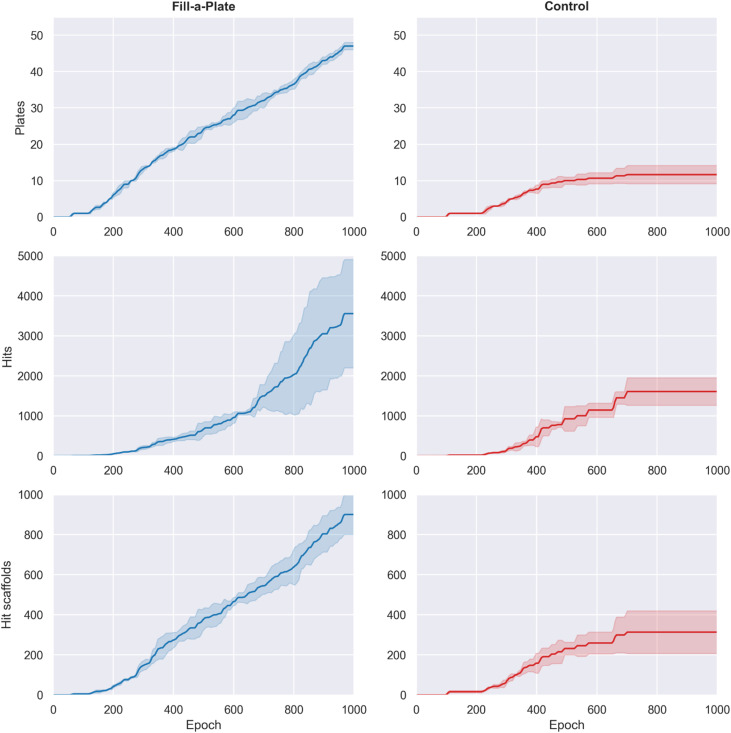
Effect of the Fill-a-Plate reward on generative behavior compared to the control. Top row shows the cumulative number of filled virtual synthesis plates across training with Fill-a-Plate filling substantially more. The middle and bottom rows show cumulative hit and hit scaffold counts, respectively. Curves represent mean performance across three independent runs (± standard deviation). Fill-a-Plate fills significantly more plates and results in higher cumulative hits and scaffolds, indicating that sustained exploration of new synthetic routes can improve discovery outcomes.

Fill-a-Plate led to a dramatic increase in the number of filled virtual parallel synthesis plates (Fig. 7, top row). By epoch 1000, Fill-a-Plate filled 47.3 ± 1.5 plates per run, compared to 11.7 ± 2.5 for the control (∼4.1-fold increase). This confirms that Fill-a-Plate actively drives the generative model to expand into new synthetic pockets.

This broader synthetic coverage translated into substantially improved hit discovery. By the end of training, Fill-a-Plate generated 3575 ± 1344 cumulative hits, compared with 1607 ± 344 for control (∼2.2-fold increase, Fig. 7 middle row). Structural novelty followed the same trend: Fill-a-Plate produced 910 ± 106 unique hit scaffolds, whereas control yielded 313 ± 106 (∼2.9-fold increase, Fig. 7 bottom row).

Fill-a-Plate successfully drives the model to fill more synthetically coherent plates of molecules than control and unlike Route Popularity this improvement also translates into higher hit discovery. By continuously shifting generation toward under-represented routes, the model benefits from broader synthetic (and chemical) space exploration. Fill-a-Plate therefore does more than diversify synthetic routes, it translates that diversification into significantly improved hit and hit scaffold discovery.

**Fig. 8:**
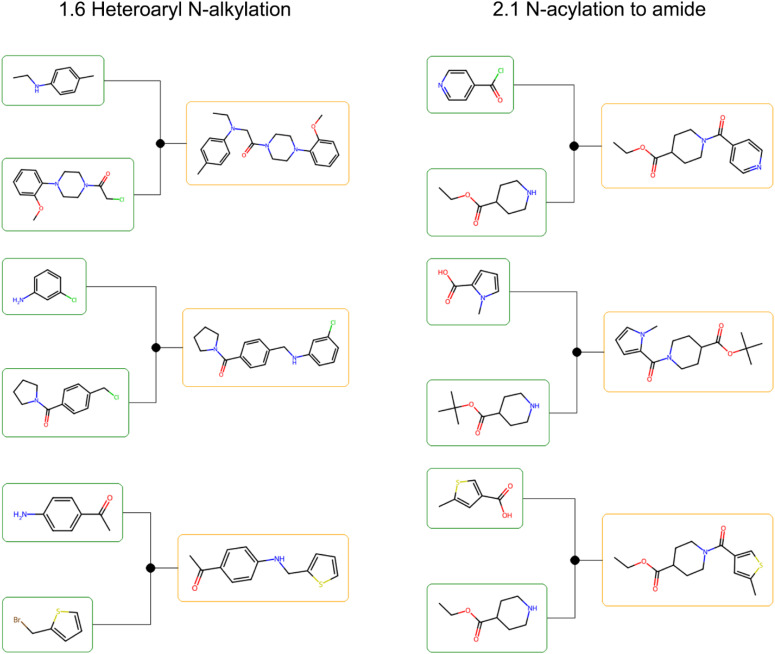
Representative retrosynthetic trees for hit molecules from two fully filled virtual synthesis plates generated under the Fill-a-Plate reward. (Left): hits accessible *via* single-step 1.6 Heteroaryl *N*-alkylation. (Right): hits formed through single-step 2.1 *N*-acylation to amide. Within each plate, hits share a consistent synthetic strategy and rely on Enamine Building Blocks starting materials, while retaining analogue-level structural variation.

To visualize the practical implications of Fill-a-Plate, we examined retrosynthetic trees for representative hits from two fully filled plates (Fig. 8). In both single-step 1.6 Heteroaryl *N*-alkylation and 2.1 *N*-acylation to amide plates, molecules follow a shared synthetic strategy using Enamine Building Blocks as starting materials, while still displaying analogue-level structural diversity.

Importantly, regardless of metric improvements, synthesis-unaware generation provides no indication of synthetic feasibility or route coherence on its own (synthetic metrics for control runs were recorded *via* SynthSense with its scoring weight set to zero). SynthSense, when active within MPO, explicitly guides the generative model toward compounds aligned with desired synthetic strategies, providing actionable insight into which molecules can realistically be synthesized.

## 4 Discussion

SynthSense represents a comprehensive approach to synthesis-aware molecular design, offering intrinsic and extrinsic reward functions. By integrating detailed retrosynthetic feedback from AiZynthFinder into the generative process, SynthSense guides molecular design by dynamically navigating the synthetic landscape.

Extrinsic reward functions provide direct synthetic feedback based on user-specified criteria, such as preferred starting materials, reactions or a reference route. These functions are particularly valuable when the design objective is well-defined and can be encoded as a set of explicit rules or preferences.

The true novelty of SynthSense lies in its intrinsic rewards, which allow real-time retrosynthetic feedback to shape the generative process itself. Route Popularity operates at the batch level, rewarding molecules that share common route signatures. This successfully concentrates design around synthetically productive routes, but our results show that strong convergence can over-exploit a narrow synthetic pocket, leading to repeated regeneration of similar chemotypes and only modest hit discovery gains. In other words, a model that learns how to make molecules does not necessarily learn which molecules become high-quality hits.

Fill-a-Plate mitigates this over-exploitation by introducing a route diversity filter, penalizing oversaturated routes and rewarding molecules that populate under-represented synthetic plates. This shifts the search strategy from exploiting a single productive synthetic niche toward continuous exploration of synthetic space. In contrast to Route Popularity, Fill-a-Plate produces significantly more hits than control, and almost three times more unique hit scaffolds, demonstrating that sustained synthetic diversification can yield meaningful discovery gains.

The complementary nature of intrinsic and extrinsic functions is a central strength of SynthSense. Extrinsic functions provide interpretability, and user control, while intrinsic functions enable the generative model to respond flexibly to emerging patterns. Together, these rewards can be used for a wide range of discovery objectives, from focused exploitation of known chemistry to broad exploration of new synthetic space.

The integration of route-level retrosynthetic analysis and reaction classification in SynthSense ensures that the feedback provided to the generative model is grounded in realistic synthetic logic, moving beyond traditional, structure-based SA scores. Its modular design also makes it simple to incorporate additional rewards, such as those reflecting cost, common intermediates, reaction conditions or any other user-defined synthetic constraints.

In conclusion, SynthSense offers a flexible way for embedding synthetic knowledge into generative molecular design. By combining intrinsic and extrinsic RL rewards, it transforms synthesizability into a controllable design parameter rather than a *post hoc* filter. The demonstrated gains in feasible hit rates, route coherence, and parallel synthesis potential highlight its practical utility for real-world discovery workflows. While Fill-a-Plate successfully prevents over-exploitation of a single synthetic niche seen in Route Popularity, its reward operates solely on retrosynthetic information and is blind to molecular quality (*e.g.*, QED, ROCS scores). As a result, it can efficiently fill a virtual plate with synthetically coherent molecules, but not all of them are hits. In the current implementation, hits within plates are identified *post hoc*. Ultimately, synthesis-aware generative models should strive to maximize both what can be synthesized and what is worth synthesizing, enabling closed-loop molecular discovery that is not only synthetically practical but also genuinely productive.

## Author contributions

A. E. created the initial prototype, and D. D. and A. V. further developed the concept, code and wrote the manuscript. J. P. J. contributed to the manuscript, experimental design, and analysis of results. Other authors tested the code, and all authors read and approved the final manuscript.

## Conflicts of interest

H.H. Loeffler, A. Voronov and J. P. Janet are employees of, and potentially hold shares in, AstraZeneca.

## Supplementary Material

SC-OLF-D5SC09263A-s001

## Data Availability

SynthSense is licensed under the Apache 2.0 license. It is free to use and modify, which requires one to retain the original copyright notice. Source code and example data are available at https://github.com/raweru/synthsense. SynthSense is also available by default in REINVENT version 4.7 and newer. Supplementary information (SI) is available. See DOI: https://doi.org/10.1039/d5sc09263a.
