# Supplementary material for: Synthesizability via reward engineering: expanding generative molecular design into synthetic space
Source: Chem Sci. 2026 Apr 7;17(20):10015–28. doi: 10.1039/d5sc09263a (PMC13054889; doi:10.1039/d5sc09263a)
Supplement: SC-017-D5SC09263A-s001 [file SC-017-D5SC09263A-s001.pdf]

Supplementary Information (SI) for Chemical Science.
This journal is © The Royal Society of Chemistry 2026

Synthesizability via Reward Engineering: Expanding Generative Molecular Design into Synthetic Space

Dominik Dekleva* †§, Alexey Voronov* ‡, Jon Paul Janet ‡, Albin Ekborg ‡⊥, Jure Borišek †, Martina H. Rambaher §, Hannes H. Loeffler ‡

† National Institute of Chemistry, Ljubljana, Slovenia

§ Faculty of Pharmacy, University of Ljubljana, Ljubljana, Slovenia

‡ Molecular AI, Discovery Sciences, BioPharmaceuticals R&D, AstraZeneca, Gothenburg, Sweden

⊥ Department of Physics, Chalmers University of Technology, Gothenburg, Sweden

1 ROCS Setup

Rapid Overlay of Chemical Structures (ROCS) was used as a ligand-based reward function to evaluate 3D similarity between generated molecules and a reference ligand. The method assesses both molecular shape and chemical feature overlap by representing atoms as Gaussian functions and calculating their shared volume.

Shape similarity is complemented by "color" similarity, which captures pharmacophoric features such as hydrogen bond donors/acceptors, charged groups, hydrophobic moieties, and aromatic rings, assigned using the Implicit Mills Dean force field [28]. The Tanimoto Combo Score in REINVENT summarizes the overlap of shape and color between two molecules with a score between 0–1, the average of both components as opposed to the standard ROCS implementation which adds these scores together, ranging between 0 and 2. As the reference, we selected the COX-2 inhibitor SC-558 (Fig. S1, PDB ID: 1CX2). A ROCS query was prepared from its crystallographic binding pose, with the following color features used: two ring features for the aromatic rings and one hydrogen bond acceptor/donor pair for the primary sulfonamide group. Conformer ensembles for each molecule were generated using OpenEye's OMEGA [29, 30] with default stereochemistry disabled, a maximum of 200 conformers per ligand, and an energy window of 10 kJ mol-1. Under these conditions, the self-overlay of SC-558 yielded a Tanimoto Combo score of 0.6, which we used as the similarity threshold for identifying hits.

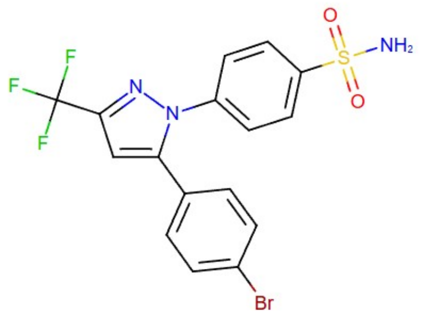

Figure S1: Structure of COX-2 inhibitor SC-558, used as reference ligand for ROCS.

2 Reference route hit scaffold plot

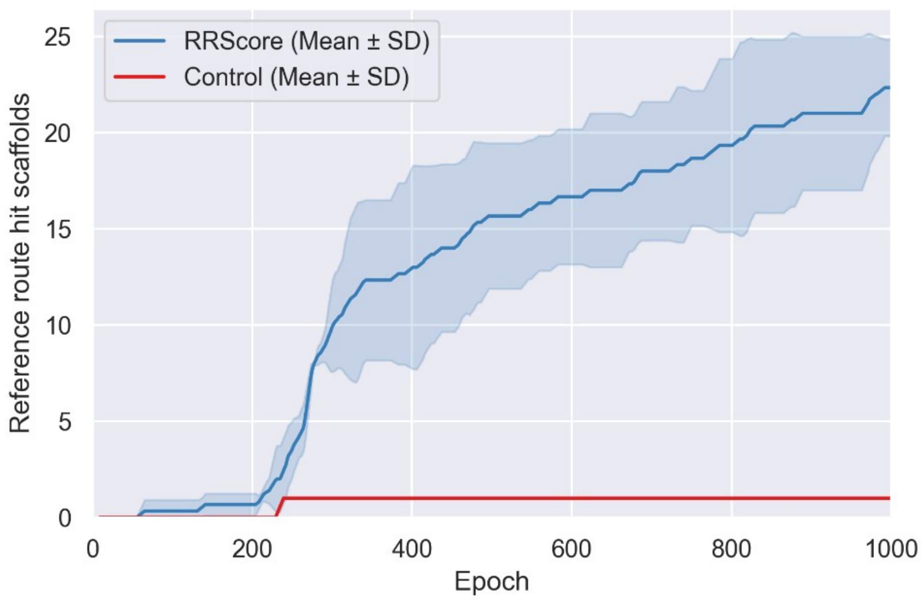

Figure S2: Training progression with the RRScore reward compared to the control. Plot shows the accumulation of reference route hit scaffolds. Each curve represents the mean across three independent runs (± standard deviation). RRScore generated significantly more reference route hit scaffolds than the control.

3 Multi-Parameter Optimization (MPO) plots

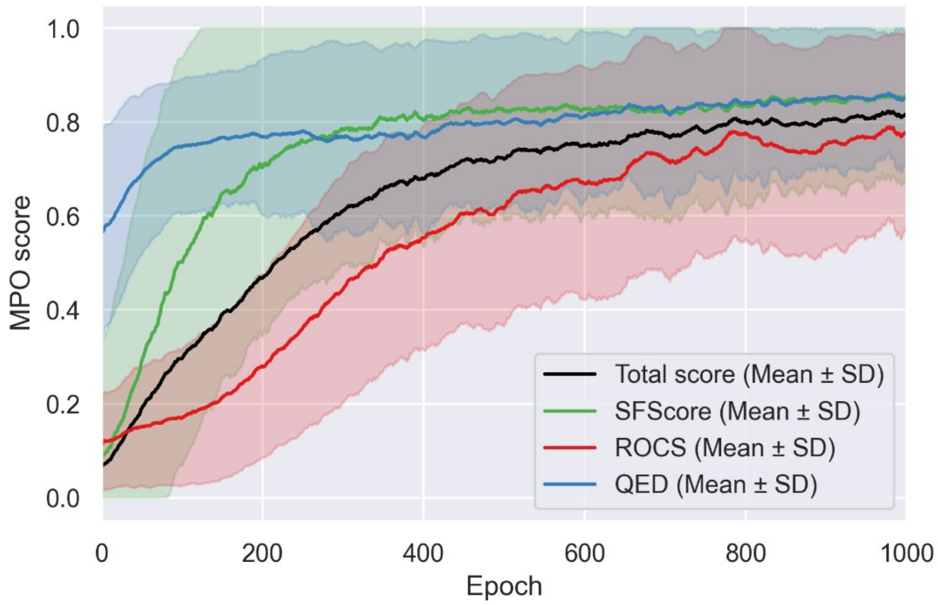

Figure S3: MPO scores for reward functions during SFScore training. Mean ± SD across three runs for QED (blue), ROCS similarity (red), SFScore (green), and total MPO score (black). QED stabilizes early, ROCS increases steadily, and SFScore converging fast within 200 epochs.

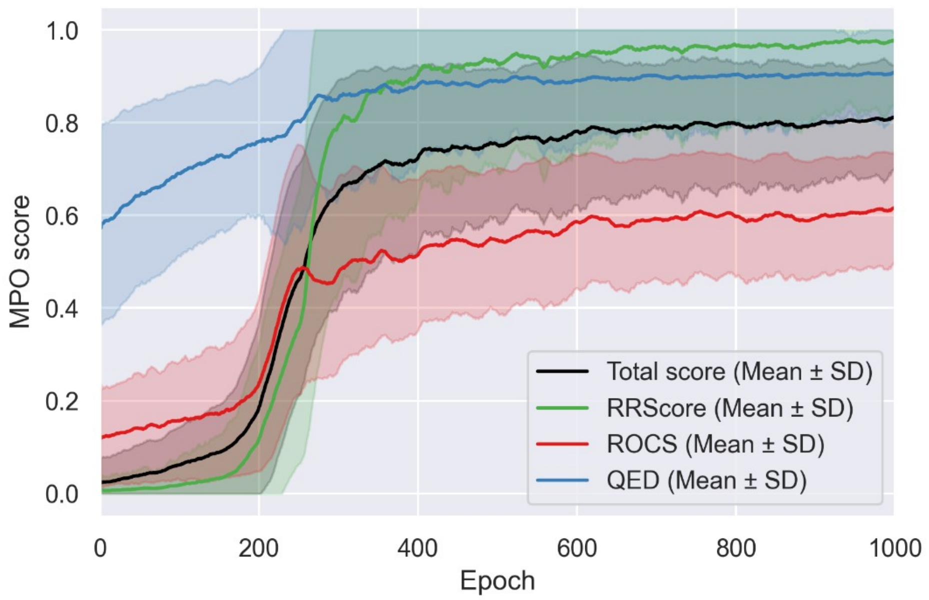

Figure S4: MPO scores for reward functions during RRScore training. Mean ± SD across three runs for QED (blue), ROCS similarity (red), RRScore (green), and total MPO score (black). QED stabilizes early, ROCS and RRScore converging fast within 300 epochs.

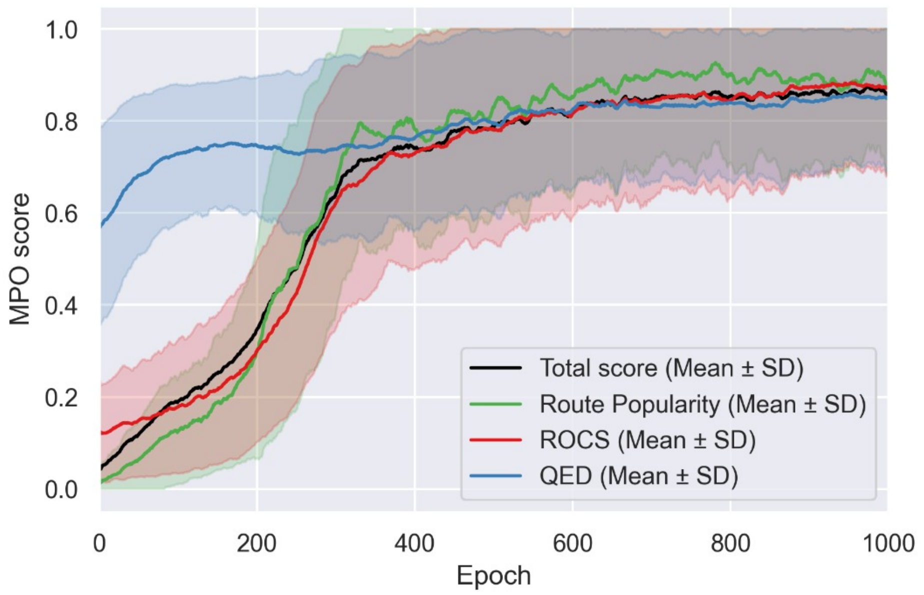

Figure S5: MPO scores for reward functions during Route Popularity training. Mean ± SD across three runs for QED (blue), ROCS similarity (red), Route Popularity (green), and total MPO score (black). QED stabilizes early, ROCS and Route Popularity converging fast within 400 epochs.

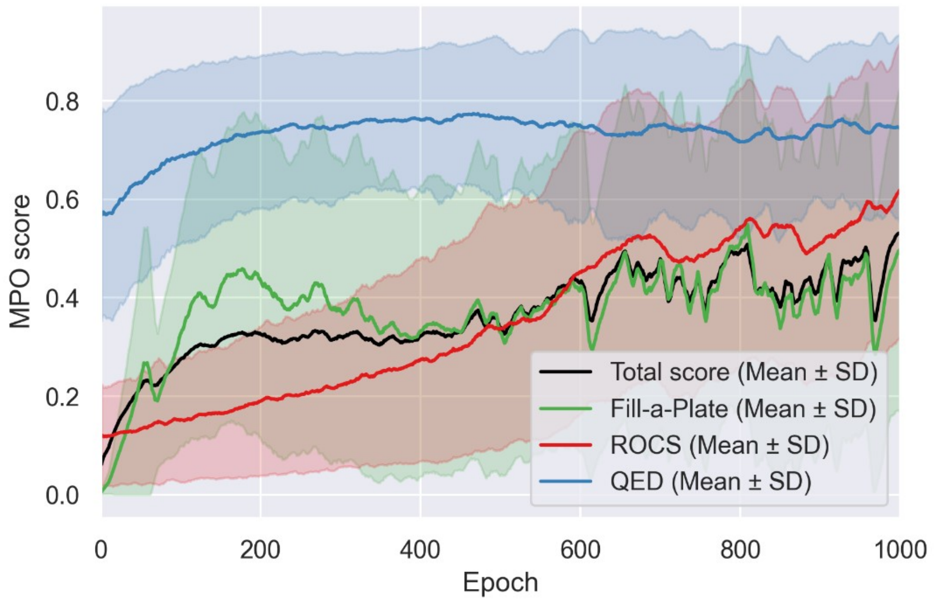

Figure S6: MPO scores for reward functions during Fill-a-Plate training. Mean ± SD across three runs for QED (blue), ROCS similarity (red), Fill-a-Plate (green), and total MPO score (black). QED stabilizes early, ROCS increases steadily, and Fill-a-Plate fluctuates as plates are filled.

4 Chemical diversity plots

Chemical (scaffold) diversity was quantified per epoch as the fraction of unique Bemis-Murcko scaffolds relative to the total number of molecules in the batch, averaged across replicates.

1.0 - every molecule in the batch has a unique scaffold, maximum diversity

0.1 - only 10% of molecules have distinct scaffolds, meaning 90% share the same core framework

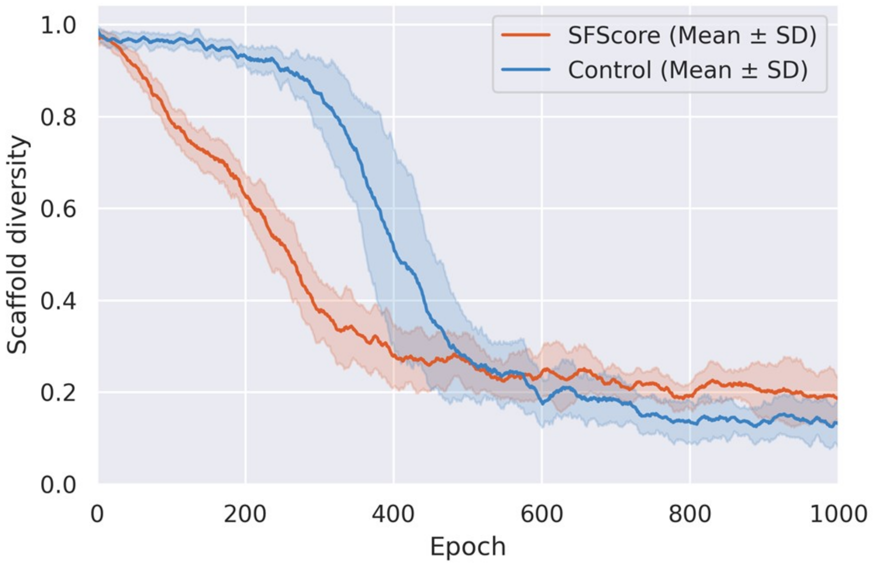

Figure S7: Scaffold diversity during SFScore training. Mean ± SD across three runs for SFScore (orange) and control (blue). The control maintains higher scaffold diversity during early training, followed by a sharp decline around mid-training, whereas SFScore shows a steadier reduction in diversity and stabilizes at a slightly higher level in later epochs.

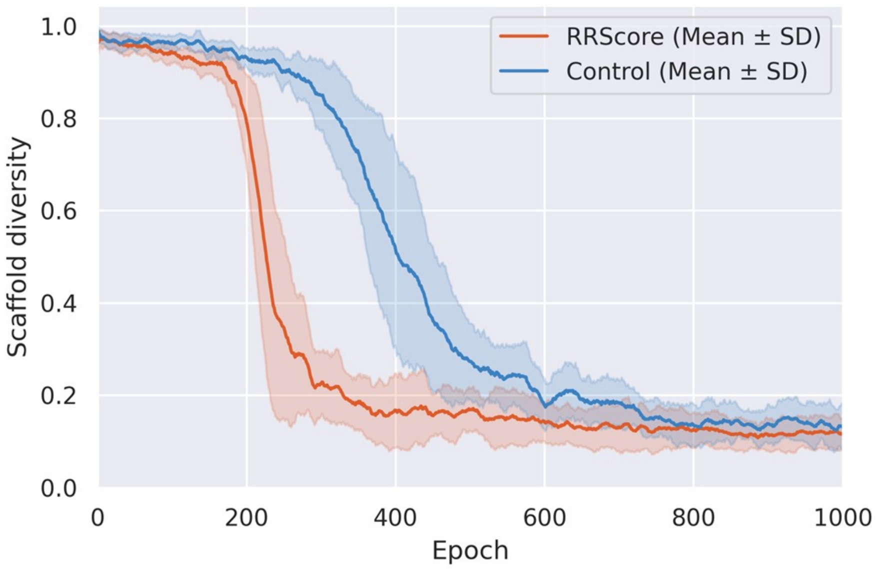

Figure S8: Scaffold diversity during RRScore training. Mean ± SD across three runs for the RRScore (orange) and control (blue). RRScore shows a rapid decline in scaffold diversity early in training, indicating faster convergence to a narrower set of scaffolds, whereas the control maintains higher diversity for longer before gradually decreasing over later epochs.

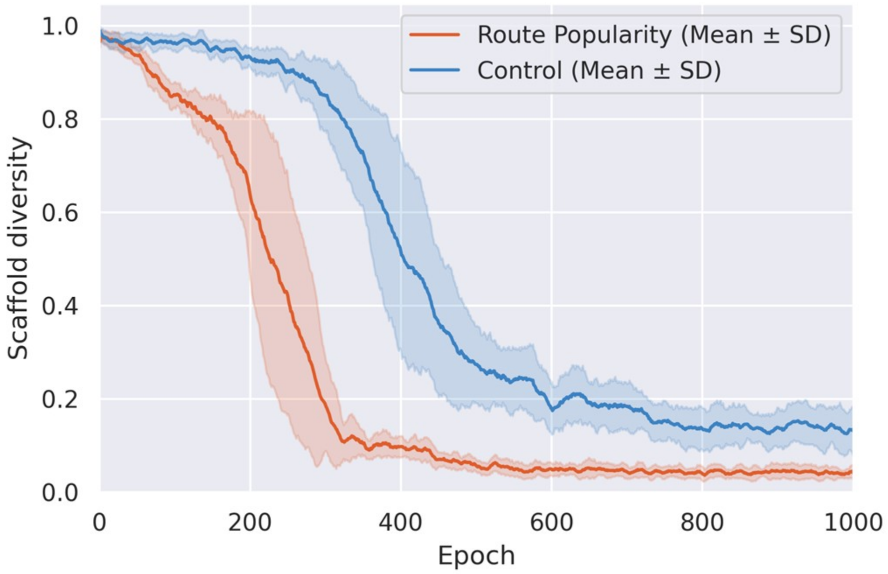

Figure S9: Scaffold diversity during Route Popularity training. Mean ± SD across three runs for the Route Popularity (orange) and control (blue). Route Popularity leads to a rapid loss of scaffold diversity early in training, indicating strong convergence toward a limited set of scaffolds, whereas the control maintains higher diversity for longer before gradually decreasing over later epochs.

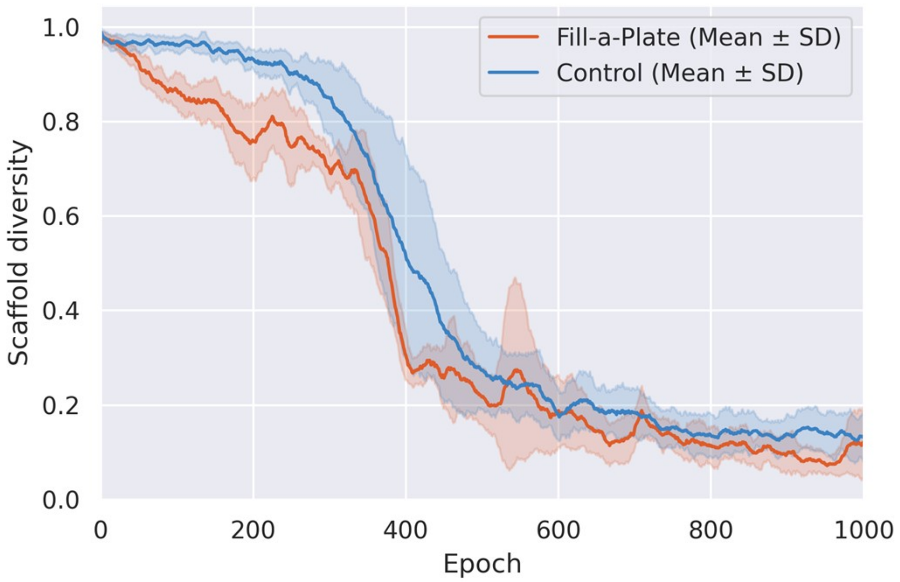

Figure S10: Scaffold diversity during Fill-a-Plate training. Mean ± SD across three runs for the Fill-a-Plate (orange) and control (blue). Fill-a-Plate shows a gradual reduction in scaffold diversity during early training followed by fluctuations as training progresses, while the control maintains higher diversity for longer before steadily decreasing in later epochs.
